# Transition from Simple V-V to V-A and Hybrid ECMO Configurations in COVID-19 ARDS

**DOI:** 10.3390/membranes11060434

**Published:** 2021-06-09

**Authors:** Piotr Suwalski, Jakub Staromłyński, Jakub Brączkowski, Maciej Bartczak, Silvia Mariani, Dominik Drobiński, Konstanty Szułdrzyński, Radosław Smoczyński, Marzena Franczyk, Wojciech Sarnowski, Agnieszka Gajewska, Anna Witkowska, Waldemar Wierzba, Artur Zaczyński, Zbigniew Król, Ewa Olek, Michał Pasierski, Justine Mafalda Ravaux, Maria Elena de Piero, Roberto Lorusso, Mariusz Kowalewski

**Affiliations:** 1 Department of Cardiac Surgery, Central Clinical Hospital of the Ministry of Interior, Centre of Postgraduate Medical Education, 02-507 Warsaw, Poland; suwalski.piotr@gmail.com (P.S.); jakubstaromlynski@gmail.com (J.S.); kuba.braczkowski@gmail.com (J.B.); mcjbrtczk@gmail.com (M.B.); dominik.drobinski@gmail.com (D.D.); radek.sm@gmail.com (R.S.); marzena.franczyk@cskmswia.pl (M.F.); wojciech.sarnowski@cskmswia.pl (W.S.); agnieszka.gajewska@cskmswia.pl (A.G.); vera@rallywitkowscy.pl (A.W.); ewa.olek.98@wp.pl (E.O.); michalpasierski@gmail.com (M.P.); 2 Cardio-Thoracic Surgery Department, Heart and Vascular Centre, Maastricht University Medical Centre, 6229 HX Maastricht, The Netherlands; s.mariani1985@gmail.com (S.M.); jmravaux@hotmail.com (J.M.R.); marieledep@gmail.com (M.E.d.P.); roberto.lorussobs@gmail.com (R.L.); 3 Department of Anesthesiology and Intensive Care, Central Clinical Hospital of the Ministry of the Interior and Administration, 02-507 Warsaw, Poland; konstantys@gmail.com; 4 Central Clinical Hospital of the Ministry of the Interior and Administration, 02-507 Warsaw, Poland; wwierzba@post.pl (W.W.); artur.zaczynski@cskmswia.pl (A.Z.); zbigniew.krol@cskmswia.pl (Z.K.); 5 Satellite Campus in Warsaw, University of Humanities and Economics in Lodz, 90-212 Warsaw, Poland; 6 Department Anaesthesia-Intensive Care, San Giovanni Bosco Hospital, 80144 Turin, Italy; 7 Thoracic Research Centre, Collegium Medicum, Nicolaus Copernicus University, Innovative Medical Forum, 87-100 Bydgoszcz, Poland

**Keywords:** extracorporeal life support, extracorporeal membrane oxygenation, acute respiratory distress syndrome, cardiogenic shock, COVID-19, SARS-CoV-2

## Abstract

In SARS-CoV-2 patients with severe acute respiratory distress syndrome (ARDS), Veno-Venous Extracorporeal Membrane Oxygenation (V-V ECMO) was shown to provide valuable treatment with reasonable survival in large multi-centre investigations. However, in some patients, conversion to modified ECMO support forms may be needed. In this single-centre retrospective registry, all consecutive patients receiving V-V ECMO between 1 March 2020 to 1 May 2021 were included and analysed. The patient cohort was divided into two groups: those who remained on V-V ECMO and those who required conversion to other modalities. Seventy-eight patients were included, with fourteen cases (18%) requiring conversions to veno-arterial (V-A) or hybrid ECMO. The reasons for the ECMO mode configuration change were inadequate drainage (35.7%), inadequate perfusion (14.3%), myocardial infarction (7.1%), hypovolemic shock (14.3%), cardiogenic shock (14.3%) and septic shock (7.1%). In multivariable analysis, the use of dobutamine (*p* = 0.007) and a shorter ICU duration (*p* = 0.047) predicted the conversion. The 30-day mortality was higher in converted patients (log-rank *p* = 0.029). Overall, only 19 patients (24.4%) survived to discharge or lung transplantation. Adverse events were more common after conversion and included renal, cardiovascular and ECMO-circuit complications. Conversion itself was not associated with mortality in the multivariable analysis. In conclusion, as many as 18% of patients undergoing V-V ECMO for COVID-19 ARDS may require conversion to advanced ECMO support.

## 1. Introduction

The use of Extracorporeal Membrane Oxygenation (ECMO) as support for adult patients diagnosed with acute respiratory distress syndrome (ARDS) has dramatically increased in the last decades and particularly during the 2009 H1N1 pandemic and the SARS-CoV-2 pandemic [1,2]. While the veno-venous (V-V) configuration represents the mainstay of ECMO therapy for refractory respiratory failure [3,4], a subset of patients might experience hemodynamic instability, inadequate drainage and perfusion or worsening cardiac dysfunction requiring conversion to other ECMO modes, mainly veno-arterial (V-A) or hybrid ECMO configurations [5,6]. 

Adults suffering from respiratory failure and requiring V-A or hybrid ECMO have historically had poor outcomes, with prohibitively high bleeding, stroke and mortality rates [7,8,9]. Some of these disparities in outcomes may be a result of the increased complexity and severity of these patients. It is, thus, mandatory to early identify ARDS patients at risk of ECMO configuration change to apply such modification in a timely manner and/or prevent catastrophic outcomes.

This is particularly true for patients diagnosed with coronavirus disease 2019 (COVID-19), a clinical manifestation of severe acute respiratory syndrome coronavirus 2 (SARS-CoV-2). Although clinical manifestations of COVID-19 are mainly respiratory, 20–25% of patients develop some degree of cardiovascular compromise [10,11,12]. When this happens in patients already supported by V-V ECMO, it might lead to cardiogenic shock, right ventricular failure, life-threatening arrhythmias or even cardiac arrest. In these cases, the V-V ECMO is not sufficient nor adequate to counteract such adverse events and a shift toward V-A or hybrid ECMO modes might be required. However, the literature is still lacking robust data on this specific patient cohort.

The aim of this study is to present an analysis from a single high-volume COVID-19 hub to describe the characteristics of COVID-19 patients requiring conversion from V-V to other ECMO configurations, the overall outcome, and to identify potential predictors for ECMO upgrades. 

## 2. Materials and Methods

### 2.1. Study Design and Patient Population

This observational retrospective study was based on our institutional registry, which prospectively included all consecutive cases of COVID-19 ECMOs admitted to the Centre of Extracorporeal Therapies (CET—Warsaw, Poland) between 1 March 2020 to 1 May 2021. The CET has been serving as third level COVID-19 reference hub and, in the same time, a subunit of the Clinical Department of Cardiac Surgery, using the expertise of cardiac anaesthetists, cardiac surgeons, cardiologists, perfusionists, and intensivists and admitting patients from the entire voivodeship and beyond. 

Recently, due to the growing number of patients requiring extracorporeal therapy, the CET has expanded and now includes the general Department of Anaesthesiology and Intensive Care (Central Clinical Hospital of the Ministry of Interior and Administration). The details on CET have been described elsewhere [13]. Adult patients were included in the registry if they were candidates for ECMO therapy and tested positive for SARS-CoV-2 Infection. 

Patients also had to have met the criteria for ECMO support in acute refractory respiratory failure (ARRF) as described by the Board of Intensive Care document and adopted by the Agency for Health Technology Assessment and Tariff System (AOTMIT) [14]. This retrospective analysis of the registry excluded patients primarily supported with V-A ECMO and other advanced support configurations. Patients requiring multiple ECMO runs and patients on ECMO at the time of the data analysis were also excluded. 

Finally, patients included in the analysis were categorized in two groups: those who remained on V-V ECMO and those who underwent conversion from V-V to other forms of support including V-A, veno-venoarterial (V-VA), venoveno-arterial (VV-A), veno-venovenous (V-VV), venoveno-venous (VV-V) or other configurations with multiple cannulations. The decision for ECMO configuration change was taken by the local ECMO heart team. Unfractionated heparin (UFH) was administered at ECMO start with an initial bolus of 50 U/kg and titrated to maintain an activated clotting time >150 s. In patients for whom major bleeding occurred, the UFH infusion was stopped. This study was approved by the Institutional Review, and patient consent for inclusion in the registry was waived.

### 2.2. End-Points and Definitions

The primary end-point was early mortality defined as mortality (of any cause) during index hospitalization or within 30-days of ECMO start. Secondary end-points were complications as identified through the application of institutional protocols and Extracorporeal Life Support Organization (ELSO) definitions [15]. 

The collected variables were grouped before analysis as follows: demographic data, comorbidities, laboratory data, medications and ECMO course data. Transesophageal echocardiography (TEE) was performed to assess ventricular function and determine the positioning of the ECMO cannulas. All patients underwent computed tomography to determine the extent of pulmonary involvement. 

### 2.3. Statistical Analysis

Demographic and clinical variables are expressed as a count (with percentage) for categorical variables and the mean (±standard deviation) or median (interquartile range, IQR) for continuous variables after evaluation for normality. Group comparisons were made using the Mann–Whitney U test where appropriate for the continuous variables and Pearson’s χ^2^ or Fisher’s exact test for categorical variables. A two-tailed *p*-value of <0.05 was considered significant. To evaluate the risk predictors of in-hospital mortality, variables that achieved a *p* value of less than 0.2 in the univariable analysis were examined using multivariable analysis with forward stepwise logistic regression. 

The following pre-ECMO variables were included in the multivariable analysis: age, weight, days of mechanical ventilation, mean airway pressure, pH, serum bicarbonate, mean arterial pressure, year of ECMO, pre-ECMO disease and conditions, on-ECMO drugs and interventions, and on-ECMO complications. Complications occurring in both groups are reported as a number (%) with the corresponding odds ratios (ORs) and 95% CIs. Analyses were performed using SPSS 26.0 (IBM, Armonk, NY, USA).

## 3. Results

**Figure 1:**
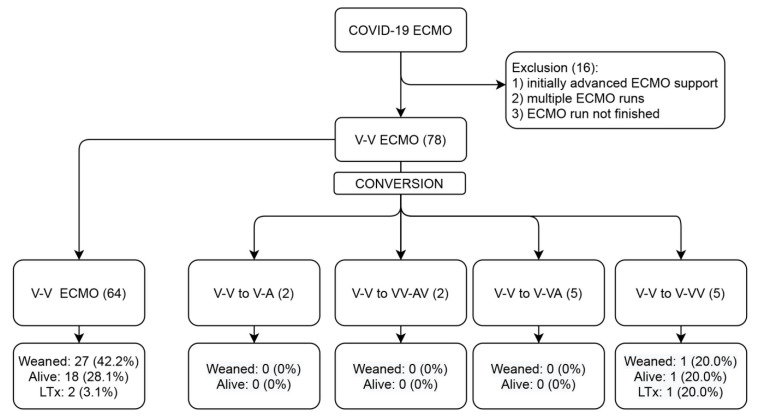
Study flow diagram. ECMO, extracorporeal membrane oxygenation; V, venous; A, arterial; and LTx, lung transplantation.

Figure 1 represents the patient flow during the study process. During the course of the study, 78 patients underwent V-V ECMO therapy for COVID-19-induced ARDS, which constituted 5.5% of all 1409 COVID-19 ARDS ICU admissions. Fourteen (18%) of those required conversions to modified forms of ECMO support for the following reasons: inadequate drainage (35.7%), inadequate perfusion (14.3%), acute myocardial infarction (AMI) (7.1%), hypovolemic shock (14.3%), cardiogenic shock (14.3%) and septic shock (7.1%). The baseline characteristics of the two groups are listed in Table 1. Patients undergoing transition to modified ECMO support were younger and less often had comorbidities but none of the differences reached statistical significance. 

Table 2 lists ECMO therapy-related details. There were no differences between patients who subsequently underwent conversion and those who remained on V-V ECMO with respect to the initial ECMO variables, except for the use of dobutamine, which was required more often in the conversion group (10.9% vs. 35.7%; *p* = 0.023). The median pro-BNP values during the first 24 h were 395 pg/mL (IQR: 184–1855) in the V-V ECMO group and 909 pg/mL (IQR: 478–6354) in the conversion group but with no significant differences (*p* = 0.209). 

The mean time to conversion was 6.5 days. In the univariable analysis, none of the variables assessed (Appendix A—Table A1) were significantly predictive of conversion to other forms of ECMO therapy. In multivariable analysis, however, the use of dobutamine (*p* = 0.007) and a shorter ICU duration (*p* = 0.047) predicted conversion. When limited to arterial conversion mode (V-V to V-A, V-V to VV-AV and V-V to V-VA), a shorter ICU time, younger age, shorter time on ECMO and lower BMI and BSA, together with higher FiO_2_, ferritine and alanine transaminase were predictive of conversion in the univariable analysis as well.

### Clinical Outcomes

**Figure 2:**
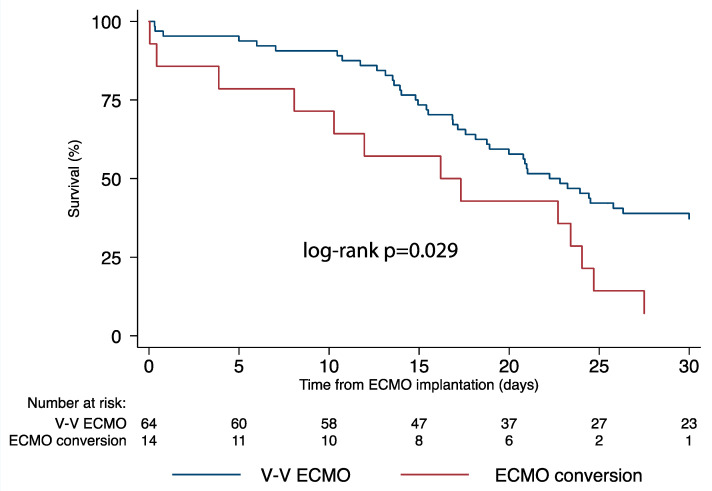
Survival comparison between veno-venous ECMO and more advanced ECMO support configuration forms. ECMO, extracorporeal membrane oxygenation.

Nineteen patients (24.4%) survived to discharge or lung transplantation; the 30-day mortality was higher in patients who underwent conversion to more advanced ECMO configurations (Figure 2) (Log-rank *p* = 0.029). Table 3 lists the complications occurring during ECMO therapy. Major bleeding was most common complication (67.9%), followed by sepsis (42.3%), continuous veno-venous hemofiltration (30.8%) and multiorgan failure (20.5%). In patients who did not undergo conversion, the odds of cardiovascular complications (OR [95% CIs]: 0.16 [0.05–0.58]; *p* = 0.005), limb complications (OR [95% CIs]: 0.06 [0.01–0.61]; *p* = 0.018) and circuit complications (OR [95% CIs]: 0.04 [0.00–0.86]; *p* = 0.040) were significantly lower compared with those transitioned to modified forms of support.

## 4. Discussion

The current study reports the characteristics and outcomes of COVID-19 patients requiring V-V ECMO and the subsequent conversion to other ECMO configurations in a single high-volume COVID-19 hub in Poland. Seventy-eight COVID-19 patients required V-V ECMO for ARDS from March 2020 to May 2021. In 18% of them, ECMO conversion was performed for inadequate drainage, inadequate perfusion, AMI, hypovolemic shock, cardiogenic shock or septic shock. Patients requiring subsequent conversion more often were treated with dobutamine already in the first 24 h of V-V ECMO support. Among the ECMO settings chosen for conversion, cardio-circulatory support was required in 64% of cases and was associated with a 100% mortality. Predictors for conversion to a different ECMO configuration were dobutamine use and a shorter ICU time. Overall, mortality and complications were significantly higher in the conversion group.

Extracorporeal membrane oxygenation is a technology that can help critically ill patients who have failed to respond to traditional care by supporting their weakened cardiovascular and pulmonary systems individually or in combination [16,17,18,19,20,21,22,23]. ECMO is typically used in the setting of isolated respiratory failure due to refractory ARDS [24]. Since March 2020 and the beginning of the SARS-CoV-2 pandemic, V-V ECMO has also become a treatment tool for critically ill patients diagnosed with COVID-19-induced ARRF. 

Indeed, ECMO may be indicated in COVID-19 patients with extreme pneumonia and acute respiratory compromise who have failed to respond to standard treatment options such as pronation, standard lung protective ventilation techniques, volume optimization and neuromuscular blockade [21,25,26]. In the case of PaO2/FiO2 < 100 mm Hg and/or arterial blood pH < 7.2 and PaCO2 > 60 mm Hg, ECMO is indicated [4]. Early V-V ECMO implantation in respiratory distress was shown to reduce respiratory-driven pressure, reduce pulmonary and systemic inflammation and improve extreme multi-organ system dysfunction [27,28]. This demonstrates the usefulness of V-V ECMO in COVID-19 patients. 

It is increasingly recognized, however, that some COVID-19 patients already treated for ARRF, may also develop combined cardiac involvement and circulatory compromise. Indeed, up to 20–25% of COVID-19 patients develop some degree of cardiovascular damage, which adversely affects their prognosis [10,11,12]. It has been demonstrated that COVID-19 patients can develop myocardial injury through direct cardiotoxicity, microvascular thrombosis and endothelial injury, pulmonary embolism, immune dysregulation, and myocarditis and myocardial infarction type 1 or 2 [10]. 

The above-mentioned conditions may, in turn, necessitate modification of the ECMO support, requiring the implantation of veno-arterial (V-A) ECMO or other ECMO modalities for primary or combined cardio-circulatory support [29,30]. Furthermore, hypovolemic or septic shock may also occur in these patients with the consequent need for adjustment of the primary ECMO configuration [11]. Finally, other circumstances, such as access- or site-related complications, differential oxygenation or vascular site bleeding, can necessitate a change in ECMO configuration by inserting an additional cannula(s), to respond to the patient’s metabolic needs and oxygenation by increasing the drainage and perfusion flow. 

**Figure 3:**
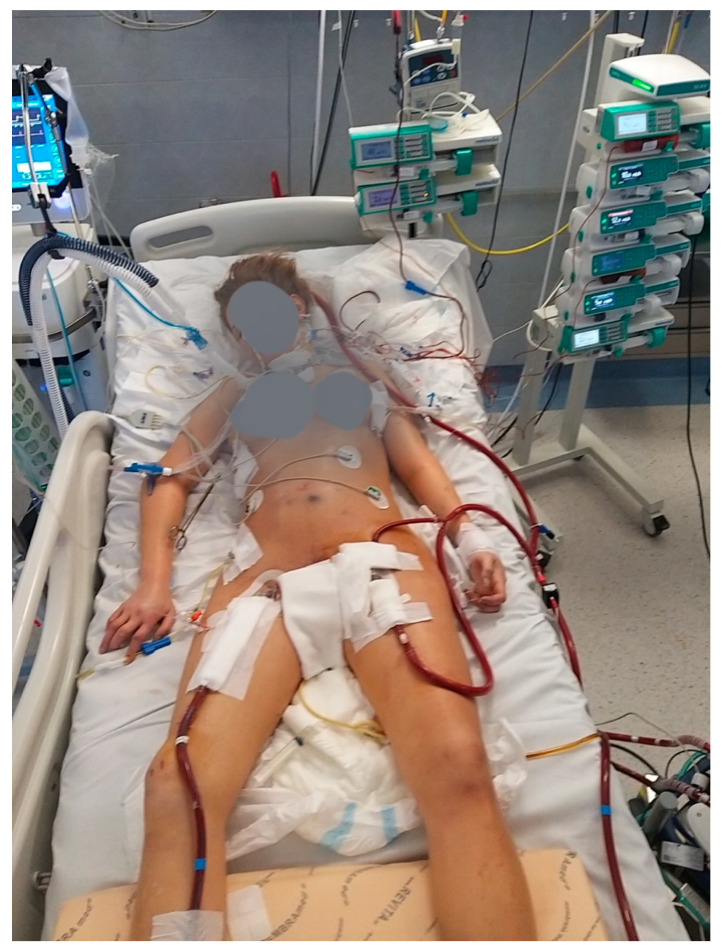
Hybrid ECMO setting for COVID-19 ARRF. V-VA (+) configuration: venous drainage femoral cannula, venous inflow jugular cannula, arterial femoral cannula and (+): femoral distal perfusion cannula.

Nevertheless, reports on ECMO configuration changes in COVID-19 patients are lacking and no clear indications exist to early identify patients at risk of ECMO conversion due to worsening of their clinical situation. It is not clear if an early conversion or the use of advanced ECMO configurations (Figure 3) since the very beginning might help to improve the survival of these patients. 

The current report, to our knowledge, is the first to address ECMO configuration changes during treatment of COVID-19 induced ARDS. Based on our experience, the need for ECMO conversion may occur in as many as 18% of V-V ECMO patients. In this population, circulatory support is required in up to 64% of patients, while the remaining cases might benefit from additional venous cannulations to improve the blood return or perfusion in respiratory support. Contrarily, the available literature reports a much lower percentage of ECMO configuration conversions ranging from 2% to 7% [31,32,33,34,35,36]. This difference might be explained with the different policies applied in ECMO centres all over the world. 

The use of advanced ECMO configurations and the practice of ECMO configuration changes are generally considered high-risk procedures, burdened with high mortality and complication risks. It is, therefore, understandable that many centres still consider this practice as the last possible choice for very complicated patients, and they refrain from using it when considered futile. Moreover, the COVID-19 pandemic has raised the important problem of resource allocation, which might have pushed many centres to avoid the use of complex ECMO configurations in patients with predicted poor outcomes [37]. It is, thus, possible to hypothesize that there could have been many more COVID-19 patients requiring an ECMO configuration change compared with those who actually received this treatment based on the abovementioned reasons. 

The alarming mortality rates that we observed in this study confirm the poor outcomes of these patients. Indeed, only one patient survived (7%) among those who underwent ECMO conversion with a significant 30-day higher mortality in the conversion group (*p* = 0.029). Moreover, complications were significantly more frequent after ECMO configuration changes; in fact, the odds of cardiovascular complications, limb complications and circuit complications were much lower in patients requiring only V-V ECMO. This can be explained considering the underlying status of the patients and the higher complexity of the ECMO circuit, irrespective of the indications to the modifications. 

Undeniably, the risk of complications is associated with the number of cannulation sites, which are multiple in the hybrid ECMO configurations. Moreover, a considerable number of patients requiring additional drainage cannula may reflect the large extent of lung involvement and marginal oxygenation as the expression of a more advanced ARDS status. This status can manifest also with a greater extent of the inflammatory response and cytokine storm, which is a well-described process in COVID-19 patients [38].

On the other hand, the question to be asked is if these outcomes could be improved through an early identification of patients at risk and an early initiation of V-A or hybrid ECMO configurations. The reasons for conversion observed in this study were multifactorial and included inadequate drainage, inadequate perfusion and myocardial infarction followed by hypovolemic-, cardiogenic- and septic shock. As previously discussed, the cardiac involvement in COVID-19 has been highlighted in several studies although it is still partially underestimated in clinical practice as reflected by the recent guidelines [39].

We observed that patients requiring subsequent conversion were treated more often with dobutamine in the first hours of V-V ECMO support. Dobutamine is rarely used as the first inotropic and vasoconstrictive agent in such scenarios. Conversely, this may reflect the deteriorating patient status who may, at this time, require two or three agents to maintain proper organ perfusion. Moreover, median pro-BNP values during the first 24 h were 395 pg/mL (IQR: 184–1855) in the V-V ECMO group and 909 pg/mL (IQR: 478–6354) in the conversion group but with no significant differences (*p* = 0.209). We could not establish any other single reliable factor that would predict the need to convert a patient in the course of ECMO treatment to a more advanced form of support based on pre-COVID variables and/or initial ECMO laboratory values. 

To better account for the inherent differences between patients necessitating a transition to arterial ECMO modes (V-A, V-VA, VV-AV etc.) for predominantly cardiocirculatory collapse rather than just inadequate drainage (V-VV), we conducted uni/multivariable analyses again focusing only on the earlier group. The above findings can be explained, only partially, by the small sample size. In fact, ECMO is a dynamic process and so are the changes in pulmonary and cardiovascular systems during COVID-19 [40]. Further studies with larger cohorts of patients are, thus, required to investigate this topic and identify the predictors for ECMO configuration changes.

### Limitations

Certain limitations to the current analysis need to be acknowledged. First of all, this is a retrospective analysis that included patients infected with SARS-CoV-2 during the first, second and third wave of the pandemic. Therefore, we cannot exclude an effect of different viral penetration and different concomitant therapies on mortality and disease severity. In fact, in another study, we reported higher mortality rates with COVID ECMO in the second and third wave of the pandemic as compared to the first wave [13]. 

The above may be attributable, in part, to novel strains of COVID-19, in particular B1.1.7 “Kent” variant, leading to higher infection rates, more severe manifestation and more COVID-19 hospitalizations and deaths among younger individuals. Moreover, several patients received V-V ECMO implantation in peripheral hospitals and were subsequently transferred to our centre, potentially increasing the risks of complications related to the transport of unstable patients. This might have pushed some clinicians to start ECMO support with the simpler V-V configuration and later switch to more complex but necessary hybrid ECMO settings. 

Additionally, selection bias arising from the paucity of ECMO devices in the peak of the third wave as well as selection bias due to the learning curve and switching to hybrid cannulation earlier in the more recent phase cannot be excluded but is subject to another ongoing investigation. In addition, certain clinical variables e.g., time from positive testing to ECMO or the left ventricle ejection fraction are largely missing. Finally, patients who underwent multiple ECMO runs and direct V-A or hybrid ECMO support were excluded from this analysis. We cannot exclude a lower mortality in these groups and a broader analysis is required to address this question.

## 5. Conclusions

As many as 18% of patients undergoing V-V ECMO for COVID-19 ARRF may re-quire conversion to V-A or more advanced ECMO support with a high mortality and complication rate. Veno-arterial and hybrid configurations can answer the immediate needs of a patient with concurrent cardiocirculatory collapse or inadequate venous drainage. Early recognition and treatment of such conditions needs to be further investigated to improve the outcomes in such complex patients. 

## Figures and Tables

**Table 1:** Baseline patient characteristics.

Variable	Total (78)	V-V ECMO (64)	ECMO Conversion (14)	*p* Value
Age	47.0 ± 11.3	48.3 ± 10.0	44.6 ± 12.2	0.228
Female	18 (23.1%)	15 (23.4%)	3 (21.4)	0.872
BMI (kg/m^2^)	31.3 ± 9.5	32.6 ± 8.6	30.6 ± 10.5	0.449
BSA (m^2^)	2.1 ± 0.5	2.2 ± 0.4	2.1 ± 0.6	0.442
Hypertension	26 (33.3%)	22 (34.4%)	4 (28.6%)	0.677
Diabetes	13 (16.7%)	11 (17.2%)	2 (14.3%)	0.792
Smoking	5 (6.4%)	5 (7.8%)	0 (0%)	0.506
CKD	5 (6.4%)	4 (6.3%)	1 (7.1%)	0.902
CAD	1 (1.3%)	1 (1.6%)	0 (0%)	0.819
Previous MI	3 (3.8%)	3 (4.7%)	0 (0%)	0.744
HF	3 (3.8%)	3 (4.7%)	0 (0%)	0.744
SOFA	8.5 ± 3.2	8.4 ± 3.3	8.6 ± 2.9	0.834
SAPS II	35.3 ± 11.0	35.8 ± 10.7	32.9 ± 12.6	0.375
APACHE II	14.4 ± 6.6	13.9 ± 6.5	16.6 ± 6.8	0.165

V-V, veno-venous; ECMO, extracorporeal membrane oxygenation; BMI, body mass index; BSA, body surface area; CKD, chronic kidney disease; CAD, coronary artery disease; MI, myocardial infarction; HF, heart failure; SOFA, Sequential Organ Failure Assessment; SAPS II, Simplified Acute Physiology Score II; and APACHE II, Acute Physiology And Chronic Health Evaluation II.

**Table 2:** On-ECMO characteristics.

Variable	Total (78)	V-V ECMO (64)	ECMO Conversion (14)	*p* Value
Off-site implant	57 (73.1%)	49 (76.6%)	8 (51.7%)	0.141
ECMO duration (d)	16.5 ± 10.0	16.4 ± 9.4	17.8 ± 10.5	0.621
ICU duration (d)	22.3 ± 11.4	23.0 ± 11.3	22.4 ± 12.3	0.859
HLoS before ICU	6.2 ± 5.9	5.9 ± 5.8	6.6 ± 5.2	0.677
FiO_2_ (24 h)	95.1 ± 8.3	95.4 ± 7.6	93.0 ± 15.3	0.385
pH (24 h)	7.4 ± 0.1	7.4 ± 0.1	7.2 ± 1.1	0.143
paO_2_ (24 h)	60.2 ± 19.3	61.1 ± 20.1	58.5 ± 21.5	0.665
paCO_2_ (24 h)	58.8 ± 20.5	58.5 ± 20.7	59.1 ± 22.0	0.923
SpO_2_ (24 h)	86.4 ± 9.3	86.4 ± 9.7	83.9 ± 15.8	0.440
PaO_2_/FiO_2_ (24 h)	64.1 ± 22.8	64.8 ± 23.3	62.5 ± 24.3	0.740
CK (24 h)	148 (68–524)	148 (70–524)	158 (63–362)	0.292
CK-MB (24 h)	26 (17–45)	28 (18–55)	21 (17–27)	0.430
TnI (24 h)	60 (24.5–202.9)	75 (25–220)	52.8 (26.45–173.3)	0.436
proBNP (24 h)	535 (245–3105)	395 (184–1855)	909 (478–6354)	0.209
AST (24 h)	42 (28–75)	46 (30–85)	33 (28–39)	0.194
ALT (24 h)	52 (30–98)	54 (32–99)	36 (26–55)	0.155
LDH (24 h)	468 (344–908)	486 (338–1295)	455 (375–603)	0.196
Lactate (24 h)	1.6 (1.2–2.1)	1.5 (1.1–2.1)	1.8 (1.2–2.0)	0.560
Ferritin (24 h)	1852 (1426–2817)	1808 (1426–3200)	1878 (1599–2053)	0.203
IL-6	161 (49–2195)	185 (48–2626)	129 (53–488)	0.260
PT	13.4 (12.7–14.9)	13.3 (12.7–14.9)	13.4 (12.7–15.1)	0.373
APTT	38.1 (33.1–47.4)	38.1 (33.1–46.1)	37.2 (32.9–59.2)	0.165
Fibrinogen	508 (320–707)	516 (334–720)	406 (280–629)	0.688
D-dimer	4660 (2023–12,810)	4550 (2095–15,931)	4771 (1754–5403)	0.758
CRP	125.8 (61.1–194.1)	126.6 (60.5–216.8)	114.1 (87.8–165.1)	0.589
PCT	0.7 (0.2–1.9)	0.8 (0.2–1.9)	0.3 (0.2–0.8)	0.519
Hb	10.9 (9.8–12.1)	10.9 (9.9–12.5)	10.7 (9.6–11.1)	0.274
Dobutamine	12 (15.4%)	7 (10.9%)	5 (35.7%)	0.023
Dopamine	3 (3.8%)	2 (3.1%)	1 (7.1%)	0.486
Adrenaline	13 (16.7%)	11 (17.2%)	2 (14.3%)	0.792
Noradrenaline	74 (94.9%)	60 (98.3%)	14 (100%)	0.610
Atropine	5 (6.4%)	4 (6.3%)	1 (7.1%)	0.902
Levosimendan	1 (1.3%)	1 (1.6%)	0 (0%)	0.819
HR	83.2 ± 24.6	82.8 ± 21.6	83.5 ± 25.8	0.916
MAP	82.0 ± 16.1	83.6 ± 12.4	80.7 ± 19.3	0.477

ECMO, extracorporeal membrane oxygenation; ICU, intensive care unit; HLoS, hospital length of stay; FiO_2_, fraction of inspired oxygen; CK, creatinine kinase; MB, muscle-brain; TnI; I troponin; BNP, brain natriuretic peptide; AST, aspartate transaminase; ALT, alanine aminotransferase; LDH, lactate dehydrogenase; IL, interleukin; PT, prothrombin time; APTT, activated partial thromboplastin time; CRP, c-reactive protein; PCT, procalcitonin; Hb, haemoglobin; HR, heart rate; and MAP, mean arterial pressure. Numbers in parentheses are percentage or interquartile ranges where applicable.

**Table 3:** Complications.

Variable	Total (78)	V-V ECMO (64)	ECMO Conversion (14)	ORs (95% CIs)	*p* Value
Major bleeding	53 (67.9%)	45 (70.3%)	8 (57.1%)	1.78 (0.54–5.82)	0.343
Massive transfusions	7 (9.0%)	7 (10.9%)	0 (0%)	3.78 (0.2–70.14)	0.372
Circuit complications	2 (2.6%)	0 (0%)	2 (14.3%)	0.04 (0.00–0.86)	0.040
Stroke	5 (6.4%)	4 (6.3%)	1 (7.1%)	0.87 (0.09–8.40)	0.902
CVVH	24 (30.8%)	17 (26.6%)	7 (50.0%)	0.36 (0.11–1.18)	0.093
Cardiovascular	16 (20.5%)	9 (14.1%)	7 (50.0%)	0.16 (0.05–0.58)	0.005
Pulmonary	14 (17.9%)	11 (17.2%)	3 (21.4%)	0.76 (0.18–3.19)	0.709
Metabolic	7 (9.0%)	5 (7.8%)	2 (14.3%)	0.51 (0.09–2.94)	0.450
Limb	4 (5.1%)	1 (1.6%)	3 (21.4%)	0.06 (0.01–0.61)	0.018
Sepsis	33 (42.3%)	28 (43.8%)	5 (35.7%)	1.40 (0.42–4.65)	0.582
MOF	16 (20.5%)	12 (18.8%)	4 (28.6%)	0.58 (0.15–2.16)	0.414

ECMO, extracorporeal membrane oxygenation, CVVH, continuous veno-venous hemofiltration; MOF, multiorgan failure; OR, odds ratio; and CI, confidence interval.

## Data Availability

Data are available upon request.

## Appendix A

**Table A1:** Univariable and multivariable analysis. Predictors of ECMO conversion.

Variable	Univariable		Multivariable	
	OR (95% CIs)	*p* Value	OR (95% CIs)	*p* Value
Age	0.95 (0.88–1.02)	0.132	0.93 (0.85–1.03)	0.169
Female	0.73 (0.14–3.82)	0.712	-	-
BMI (kg/m^2^)	0.88 (0.26–3.04)	0.845	-	-
BSA (m^2^)	0.00 (1.15 × 10^−24^–3.67 × 10^18^)	0.804	-	-
Hypertension	0.85 (0.17–4.18)	0.841	-	-
Diabetes	1.50 (0.18–12.44)	0.705	-	-
CKD	0.97 (0.06–16.81)	0.984	-	-
SOFA	1.02 (0.84–1.23)	0.841	-	-
SAPS II	0.97 (0.91–1.04)	0.400	-	-
APACHE II	1.06 (0.97–1.16)	0.184	1.05 (0.91–1.21)	0.490
Off-site implant	0.56 (0.12–2.63)	0.462	-	-
ICU duration (d)	0.95 (0.89–1.02)	0.176	0.90 (0.82–0.99)	0.047
HLoS before ICU	1.03 (0.94–1.15)	0.456	-	-
FiO_2_ (24 h)	1.29 (0.74–2.27)	0.365	-	-
pH (24 h)	4.51 (0.00–9.26 × 10^5^)	0.809	-	-
paO_2_ (24 h)	0.74 (0.36–1.50)	0.401	-	-
paCO_2_ (24 h)	1.02 (0.95–1.10)	0.542	-	-
SpO_2_ (24 h)	1.06 (0.92–1.23)	0.402	-	-
PaO_2_/FiO_2_ (24 h)	1.26 (0.66–2.41)	0.478	-	-
CK	0.96 (0.84–1.10)	0.556	-	-
CK-MB	1.05 (0.74–1.48)	0.787	-	-
TnI	1.00 (0.99–1.01)	0.947	-	-
proBNP	1.00 (1.00–1.01)	0.263	-	-
AST	0.99 (0.98–1.01)	0.606	-	-
ALT	0.99 (0.98–1.01)	0.418	-	-
LDH	1.00 (0.99–1.01)	0.362	-	-
Lactate	0.89 (0.01–113.28)	0.964	-	-
Ferritin	1.00 (0.99–1.01)	0.729	-	-
IL-6	1.00 (1.00–1.01)	0.399	-	-
PT	0.90 (0.70–1.17)	0.440	-	-
APTT	1.01 (0.99–1.03)	0.164	1.02 (0.99–1.05)	0.149
Fibrinogen	0.99 (0.99–1.00)	0.524	-	-
D-dimer	1.00 (0.99–1.00)	0.389	-	-
CRP	0.99 (0.98–1.01)	0.748	-	-
PCT	0.99 (0.94–1.06)	0.932	-	-
Hb	0.66 (0.39–1.21)	0.193	0.59 (0.32–1.10)	0.098
Dobutamine	4.94 (0.80–30.60)	0.086	14.70 (2.11–102.20)	0.007
Dopamine	4.76 (0.15–153.17)	0.378	-	-
Adrenaline	1.62 (0.21–12.48)	0.645	-	-
Atropine	1.01 (0.06–17.25)	0.996	-	-
HR	1.02 (0.99–1.05)	0.147	1.03 (0.99–1.06)	0.086
MAP	0.97 (0.92–1.02)	0.208	-	-

Variables that presented collinearity are not presented. ECMO, extracorporeal membrane oxygenation; OR, odds ratio; CI, confidence interval; BMI, body mass index; chronic kidney disease; SOFA, Sequential Organ Failure Assessment; SAPS II, Simplified Acute Physiology Score II; APACHE II, Acute Physiology And Chronic Health Evaluation II; ICU, intensive care unit; HLoS, hospital length of stay; FiO_2_, fraction of inspired oxygen; CK, creatinine kinase; MB, muscle-brain; TnI; I troponin; BNP, brain natriuretic peptide; AST, aspartate transaminase; ALT, alanine aminotransferase; LDH, lactate dehydrogenase; IL, interleukin; PT, prothrombin time; APTT, activated partial thromboplastin time; CRP, c-reactive protein; PCT, procalcitonin; Hb, hemoglobin; HR, heart rate; and MAP, mean arterial pressure.
